# Acute idiopathic polyradiculoneuritis concurrent with acquired myasthenia gravis in a West Highland white terrier dog

**DOI:** 10.1186/s12917-016-0729-1

**Published:** 2016-06-14

**Authors:** Gabriela Dumitrita Stanciu, Gheorghe Solcan

**Affiliations:** Departament of Internal Medicine/Neurology, Faculty of Veterinary Medicine, University of Agricultural Sciences and Veterinary Medicine “Ion Ionescu de la Brad”, 8 M. Sadoveanu Alley, 700489 Iasi, Romania

**Keywords:** Electrophysiology, Dog, Acquired polyneuropathies, Postsynaptic disturbances

## Abstract

**Background:**

Acute Idiopathic Polyradiculoneuritis, an animal model for the axonal form of the Guillain – Barre Syndrome in humans and the acquired myasthenia gravis are different autoimmune disorders affecting the peripheral nerves and the neuromuscular junction, respectively. Both lead to muscle weakness and possible respiratory failure. The coexistence of these two entities combined in the same patient is rare in humans and, to our knowledge, the present case is the first reported in dogs.

**Case presentation:**

An 11-year-old West Highland WhiteTerrier female dog was referred to our clinic with a history of symmetrical weakness beginning with the pelvic limbs and evolving cranially, progressing to non-ambulatory flaccid tetraparesis over the preceding week. The history did not reveal signs of a recent other illness, trauma or exposure to a neurotoxin or raccoon bite. The last vaccination was carried out 5 months before presentation. Upon clinical examination, spinal reflexes and postural reactions were decreased in all four limbs and became absent within the following 24 h; perineal reflex was normal, and loss of voice was observed. The patient maintained its ability to urinate and defecate and it had no difficulty to eat or to drink. There were no cerebellar or sensory deficits.

The electrophysiological findings revealed positive sharp waves and complex repetitive discharges on the electromyogram, temporal dispersion of compound muscle action potentials associated with polyphasia and a slow motor nerve conduction velocity as signs of demyelination, and an increased latency of F-waves. The cerebrospinal fluid had a normal cellularity with increased protein content. A reduction by 18 % in the amplitudes of the third compound muscle action potential as compared to the first one was observed during a repetitive nerve stimulation, indicating a postsynaptic disturbance. Only the motor electrophysiology was considered in this study. The diagnosis was based on clinical and electrophysiological findings associated with a positive titer for acetylcholine receptor antibodies.

**Conclusion:**

The diagnosis of MG was based on the typical clinical findings such as dysphonia and dysphagia, decremental response of RNS and positive AChRs antibody titre. Flaccid tetraparesis associated with diminished reflexes, increase of the distal latencies and temporal dispersion which caused lower MNCV, along with the increase of the F wave latencies supported the diagnosis of AIP. A cerebrospinal fluid tap indicated an albuminocytologic dissociation sustaining the radicular implication of AIP. As such, a diagnosis of MG and AIP co-occurrence syndrome was established.

## Background

Acute idiopathic polyradiculoneuritis (AIP), an animal model for the axonal form of Landry-Guillain–Barré syndrome in humans and acquired myasthenia gravis (MG) are two autoimmune disorders affecting the peripheral nerves and the neuromuscular junction, respectively. Both lead to muscle weakness and possible respiratory failure [1].

AIP can affect man at any age with a prevalence of 1–2 cases per 100.000 people, while cases of MG can reach 15 cases per 100.000 people [2]. AIP is found worldwide in dogs, with individuals of varying ages and breeds affected. Affected dogs typically show signs of acute ascendant tetraparesis of various severity stages, and dysphonia. Facial nerve paresis is occasionally seen and in severe cases, respiratory disturbances can occur. These findings are consistent with those described in a disease known as Coonhound paralysis in areas where raccoons are endemic. The precise pathogenesis of this disease is unknown but neurological signs are associated with raccoon bites and the clinical signs can be experimentally reproduced by parenteral infusion of diseased raccoon saliva [3]. Acquired (immune-mediated) MG occurs due to the antibody mediated destruction of postsynaptic nicotinic acetylcholine receptors (AChRs).

Signs of muscular weakness may be focal with selective muscle involvement (esophageal, pharyngeal, or/and facial muscles), or diffuse, with signs of generalized muscle weakness. While the immune-mediated destruction of AChRs is well documented, the specific pathogenesis is unknown [4].

Simultaneous occurrence of the AIP and acquired MG combined in the same patientis rarely described in humans; only sixteen cases in the past 40 years were reported [5, 6] and could be explained by the involvement of (i) Autoimmunity (which seems to play an important role in the pathogenesis of both diseases); (ii) Thymoma (that can be a common cause for MG and other autoimmune neurological diseases); or (iii) Molecular mimicry between infectious agents and self-antigens which is postulated to be a trigger factor in the initiation of autoimmune diseases. This association was observed in one case with MG and GBS: anti-ganglioside antibodies (IgG) named GM1 and GQ1, and *Campylobacter jejuni* antibodies were positive. A common infectious agent could be responsible for the production of cross-reacting antibodies against myelin of peripheral nerve and AChRs, inducing GBS and MG simultaneously [7, 8].

Some molecular structures of the AChRs and the peripheral nerve may be the same or similar to each other so one kind of IgG antibody may be pathogenic to both AChR and the peripheral nerve and thus lead to the onset of MG and GBS on the same patients at the same or at a different time. To our knowledge, this is the first report of such an occurrence in the dog.

## Case presentation

An 11-year-old West Highland White Terrier female dog was referred to the Department of Clinical Sciences, Internal Medicine-Neurology of Faculty of Veterinary Medicine, University of Agricultural Sciences and Veterinary Medicine from Iași. The symptoms (symmetrical weakness beginning with the hindlimbs and evolving to forelimbs, progressing to a non-ambulatory flaccid tetraparesis) started one week before presentation. Upon clinical examination, spinal reflexes and postural reactions were decreased in all four limbs and became absent within the following 24 h, and a normal perineal reflex, and loss of voice (dysphonia) were also observed. The rectal temperature was 39.0 °C, the heart rate was 66 beats per minute and the respiratory rate was 24 per minute. The patient maintained the ability to urinate and defecate, and had no difficulty eating or drinking. No cerebellar or sensory deficits were observed. The history did not reveal signs of another recent illness (coughing, lack of appetite, vomiting, diarrhea, inactivity, or agitation), trauma or exposure to a neurotoxin or raccoon bite.

Neurological examination was followed by laboratory analyses including blood counts, serum biochemical profile (blood glucose, total proteins, albumin, blood urea nitrogen, creatinine, total bilirubin, alanine and aspartate transferase, alkaline phosphatase, creatine kinase, cholesterol and Na, P, Ca, K, Mg ions), and basic urine analyses. The results of investigated parameters were found within the reference limits of the species. At CSF examination, the albumincytologic dissociation (normal cellularity with increased protein content −50 mg of protein/dl) sustained the radicular implication of the AIP like in human patients with Landry Guillain-Barre syndrome [5].

Electrophysiological tests were carried out under sedation [4] with medetomidine hydrochloride (Domitor, Phizer, Finland) 30 μg/kg intramuscularly, using the Neuropack S, MEB 9400 K electro diagnostic System (Nihon Kohden, Japan) in the Electromyography (EMG) program for acquirement of the bioelectrical potentials. Only motor electrophysiology was considered in this study.

**Fig. 1:**
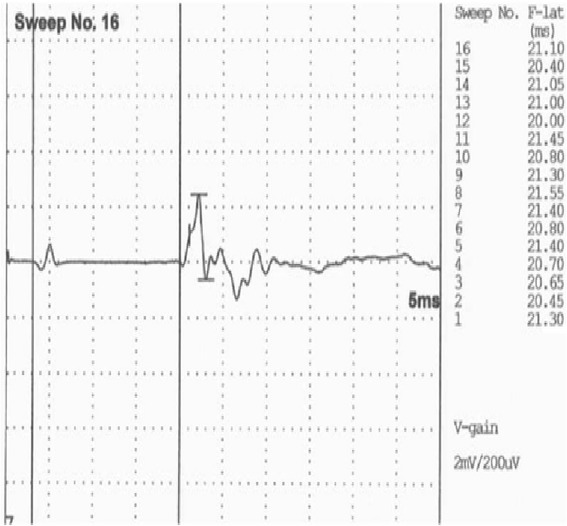
Tibial nerve F waves. Latency of 20 m/s reveal an F wave relationship significantly above the normal value (normal range <13 m/s)

Motor nerve conduction velocity (MNCV) measurements were performed on the tibial, radial, and ulnar nerved by recordings from the plantar, palmar interosseous, and extensor carpi ulnaris muscles, respectively. The electroneurography revealed a moderate reductionin MNCV of radial (45.3 vs. 72.1 ± 1.9 m/s ^[4]^) and tibial (51.4 vs 68.3 ± 4.2 m/s ^[4]^) nerves. The compound muscle action potentials (cMAPs), with a polyphasic morphology showed reduced amplitude after proximal (15 vs. 21.6 ± 1.6 mV ^[4]^) and distal (9 vs. 23.4 ± 1.5 mV ^[4]^) stimulation of the radial nerve, and after hock (9.6 vs. 23.3 ± 2.3 mV^[4]^) and hip (8.7 vs. 20.1 ± 1.6 mV ^[4]^) stimulation of the tibial nerve. Increased latency of cMAPs, after stimulation at distal segments of radial (distal: 6.5 vs. 1.3 ± 0.1 ms ^[4]^; proximal: 2.5 vs. 1.9 ± 0.1 ms ^[4]^) and tibial (hock: 13.2 vs. 2.9 ± 0.1 ms ^[4]^; hip: 8.4 vs. 6.7 ± 0.4 ms ^[4]^) nerves, was more marked. F waves could be evoked in the tibial nerve, where an increase in their latency was detected (20 ms vs <13 ms ^[4]^) –Fig. 1. The F ratio was lower than references values (F ratio at the hock stimulation = 0.21 vs. 1.75 ± 0.2 ^[4]^ and F ratio at the hip level: = 0.63 vs 0.73 ± 0.11 ^[4]^), which indicate more severe involvement of the distal part of the nerve.

**Fig. 2:**
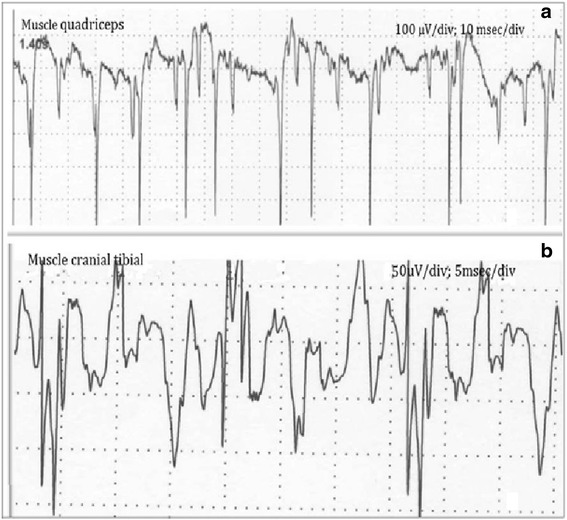
EMG traces show positive sharps waves (**a**) and atipical complex repetitive discharges (**b**)

Electromyographic examination revealed mild to moderate insertional activity and spontaneous activity consisting primarily of fibrillation potentials, positive sharp waves and complex repetitive discharges in the quadriceps, cranial tibial, extensor carpi radialis and plantar interosseous muscles (Fig. 2).

**Fig. 3:**
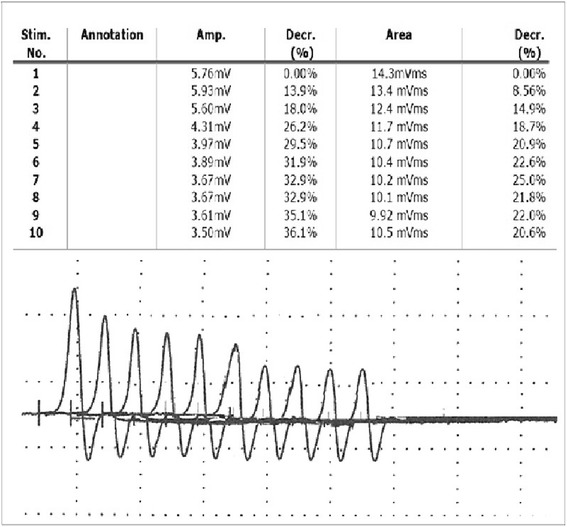
RNS test, the fatigability of the synaptic transmission. There is an obvious 18 % reduction in the amplitudes of the third cMAP compared to the first cMAP

To exclude a neuromuscular junction disease, an anti-acetylcholine receptor antibody analysis and a neural transmission evaluation by repetitive nerve stimulation (RNS) were performed using the frequency of 2 to 3 Hz. At RNS, the results revealed a reduction by 18 % in the amplitudes of the third cMAP to the first one (Fig. 3). Serum acetylcholine receptor antibody levels were highly elevated (8 nmol/L)as compared to a titre no greater than0.6 nmol/L considered by previous reports [4, 5, 9], an indicator of MG.

The fact that the EMG changes were so severe by day 7 after onset of signs would exclude tick paralysis and botulism (since both lead to signs of junctionopathy), and increased the likelihood of a neuropathy being present, in this case a radiculopathy. The tick paralysis was also excluded since no ticks were found on the animal, neither during the clinical examination, nor previously by the owner. Blood smears were prepared and stained using the May Grunwald Giemsa method and were examined under light microscopy for the presence of intracellular inclusions (or free forms) of common tick-borne pathogens; the results were negative. The tick toxin is a molecule that blocks the neuromuscular transmission within in several days by inhibiting the presynaptic membrane depolarization. Detection and removal of the ticks leads to a rapid (about 24 h) improvement of the patient’s condition [10].

A myopathy would be by far less likely due to the history, since the typical clinical picture of our patient was an ascending, areflexic paralysis, where the lower limbs were firstly affected. Within only hours to days, the weakness spread to upper limbs, while a myopathy is characterized by proximal weakness or fatigue, normal sensitivity, and later loss of reflexes only after a significant atrophy [8].

Botulism and tick paralysis were excluded within the differential diagnosis. In botulism, the clinical signs occur within 24 to 48 h after the toxin ingestion. The main symptom is a generalized flaccid paralysis which starts as a mild pelvic limb gait abnormality, then progressive tetraparesis to tetraplegia, and paralysis of the cranial musculature occur. Facial nerve paralysis, ophthalmoplegia, dysphagia, disturbances in swallowing, megaoesophagus and dysphonia can occur, too. Sensation and nociception remain normal [11]. Electrodiagnostic findings in botulism affected dogs include normal motor nerve conduction velocity, decreased M-wave amplitude, increment of the M-wave with slow/rapid supramaximal repetitive nerve stimulation and increased, "jitter", with single fiber EMG [12]. On the other hand, in tick paralysis, an ascending flaccid paralysis occurs within a few days and may cause death through respiratory paralysis. No EMG evidence of denervation was observed and the amplitude of motor potentials was markedly reduced. Nerve conduction velocity may be slightly slower than normal and terminal conduction times may be prolonged [8].

Treatment was restricted to physical rehabilitation, supportive care, and cyclosporine (4 mg/kg PO q12h) to suppress the autoimmune bases of the diseases. Starting with the second week, the patient began to make progress in its recovery and after 6 weeks it only had moderate gait disturbances. At follow up eleven months after diagnosis, the dog has no neurological deficits, and maintained on cyclosporine therapy until AChRs antibody titres reach normal physiological values. A complete set of blood tests has been performed every 3 months with the most recent AChRs antibody titre 0.8 nmol/L.

Treatment was elected to be composed of physical rehabilitation consisting of physiotherapy (massage, passive movements) and hydrotherapy (swimming), aimed at stimulating neuromuscular function and preventing early muscle atrophy, as it has been demonstrated that they are the most efficient methods of treatment for AIP in recently years [13]. Corticosteroid therapy and the more modern therapies such as plasmapheresis and intravenous immunoglobulin therapy may improve the course of the disease, but have little evidence in support of them and remain controversial [14].

In the present case, cyclosporine was associated with a positive clinical response in the treatment of acquired MG. The results are similar to those reported by Bexfield et al. [15], in a study on the management of MG using cyclosporine, after pyridostigmine bromide and prednisolone therapy proved no improvement in clinical signs.

Acquired MG is a T-cell dependent disease and cyclosporine is relatively specific for T-lymphocytes, so its use in the treatment of the acquired MG was justified [14]. When evaluating the effectiveness of immunosuppressive therapy in acquired MG, a high rate of spontaneous remission has been reported (88.7 %) by Shelton and Lindstrom [16].

Early recognition of the concurrent neuromuscular junction and peripheral nervous inflammation is a crucial factor in determining the initial treatment and eventual prognosis.

## Conclusions

The diagnosis of MG was based on the typical clinical findings such as dysphonia and dysphagia, decremental response of RNS and positive AChRs antibody titre. Flaccid tetraparesis associated with diminished reflexes, increase of the distal latencies and temporal dispersion which caused lower MNCV, along with the increase of the F wave latencies supported the diagnosis of AIP. A cerebrospinal fluid tap indicated an albuminocytologic dissociation sustaining the radicular implication of AIP. So MG and AIP concurrence syndrome was established.

## Abbreviations

AChRs, postsynaptic nicotinic acetylcholine receptors; AIP, acute idiopathic polyneuropathy; cMAP, compound muscle action potential; EMG, electromyography; MG, myasthenia gravis; MNCV, motor nerve conduction velocity; RNS, repetitive nerve stimulation
